# Pathogenesis and regenerative therapy in vitiligo and alopecia areata: focus on hair follicle

**DOI:** 10.3389/fmed.2024.1510363

**Published:** 2025-01-15

**Authors:** Yuan Zhou, Yu-Xuan Zhang, Yu-Yun Xiong, Yu-Mei Li

**Affiliations:** Department of Dermatology, Institute of Regenerative Medicine, Affiliated Hospital of Jiangsu University, Zhenjiang, China

**Keywords:** hair follicles, vitiligo, alopecia areata, autoimmune disease, clinical application

## Abstract

Vitiligo is an autoimmune disease characterized by the loss of functional melanocytes in the hair follicles and epidermis, leading to white patches on the skin and mucous membranes. Alopecia areata (AA) is a common immune-mediated condition in which autoimmune attack on hair follicles cause non-scarring hair loss. Both diseases significantly impact patients’s physical and mental health. Hair follicles, dynamic mini-organs, house diverse stem cell populations that form hair structures. Melanocyte stem cell (McSCs) and hair follicle stem cells (HFSC) located in the hair follicle bulge contribute to follicular structures during each anagen phase of the hair cycle, synchronizing periodic activities to impact color to the hair. Hair follicle dysfunction may contribute to hair loss and could potentially interfere with repigmentation efforts in vitiligo lesions. This article reviews the role of hair follicles in the pathogenesis, clinical manifestations, and therapeutic options for vitiligo and AA, aiming to deepen clinicians’ understanding of follicular involvement in these diseases and explore potential treatment avenues.

## 1 Introduction

Both vitiligo and alopecia areata (AA) are autoimmune skin disorders. Vitiligo is the most common acquired depigmenting skin condition (1), characterized by the loss of melanocytes in the epidermis and hair follicles, which results in well-defined white patches on the skin or mucous membranes (2). The global prevalence of vitiligo is esrimated to range from 0.5 to 2% (3). AA, often regarded as “a sister disease” of vitiligo, is a T-cell-mediated autoimmune disorder primarily manifesting as patchy hair loss that can progress to total alopecia. Importantly, the hair follicles in AA remain structurally intact, resulting in non-scarring hair loss (4). The global incidence of AA ranges from 0.1 to 0.2% (5).

While vitiligo and AA have traditionally been considered distinct disorders due to their varied clinical presentations and complex pathogenic mechanisms, there is evidence of a relationship between them. Approximately 1.1% of patients with AA also present with vitiligo (6). Current research on shared pathogenic mechanisms in vitiligo and AA primarily focuses on T-cell-mediated autoimmunity, elevated reactive oxygen species, and increased cellular stress levels (7, 8). However, the role of the hair follicle in the pathogenesis and treatment of both vitiligo and AA remains largely unexplored.

The hair follicle, a skin appendage containing various types of stem cells, serves as an ideal *in vivo* model for studying interactions between stem cells and their niche cells. Melanocyte stem cells (McSCs) and hair follicle stem cells (HFSCs) in the hair follicle bulge contribute to follicle structures during each anagen phase of the hair cycle, engaging in synchronized activities that impart color to the hair (9). Hair follicle dysfunction may contribute to hair loss and could potentially interfere with repigmentation efforts in vitiligo lesions (10). This study aims to use hair follicles as a common focal point to analyze and summarize the similarities and differences between vitiligo and AA, with the goal of identifying treatment methods and improving diagnostic accuracy.

## 2 Immune privilege of hair follicles in vitiligo and AA

**FIGURE 1:**
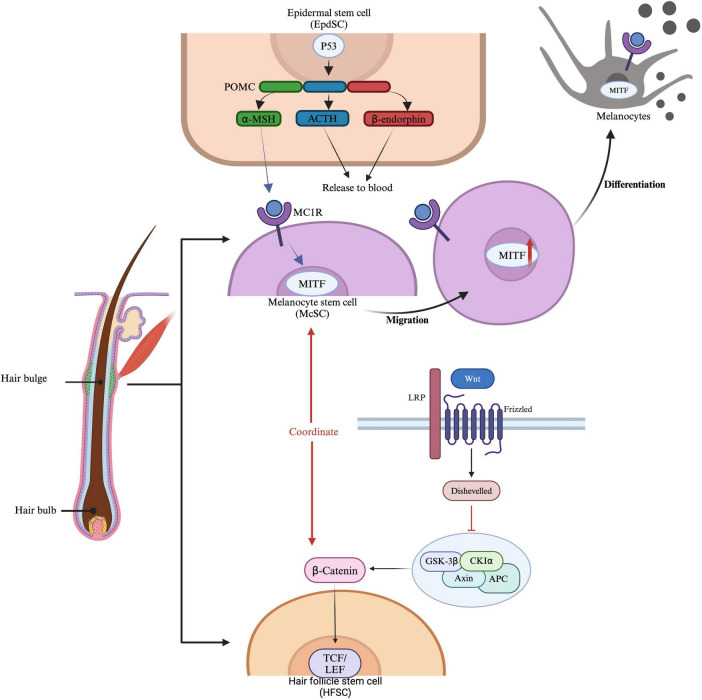
Regulation and activation of McSCs and HFSCs. Epidermal stem cells (EpdSCs) produce proopiomelanocortin (POMC), which can be further processed into α-MSH (melanocyte-stimulating hormone), ACTH (adrenocorticotropic hormone), and β-endorphin. These molecules are released into the bloodstream, where they bind to MC1R (melanocortin 1 receptor) on melanocyte stem cells (McSCs). McSCs express MITF (microphthalmia-associated transcription factor), which is essential for the development and function of melanocytes. Hair follicle stem cells (HFSCs) are influenced by β-catenin signaling, where Wnt proteins bind to receptors such as LRP and Frizzled, activating Disheveled and preventing β-catenin degradation. β-catenin then accumulates and translocates to the nucleus, where it binds to TCF/LEF transcription factors to activate gene expression. This pathway coordinates with MITF, influencing McSC migration and HFSC function, thereby promoting hair growth and pigmentation.

One of the most notable features of healthy anagen-stage hair follicles is the relative immune privilege (IP) of their epithelium, extending from the bulge region down to the hair bulb, which serves as the niche for HFSCs. McSCs, residing whthin the same bulge niche as HFSCs, undergo cyclical activation in coordination with HFSCs (Figure 1). Hair follicle regeneration follows a hierarchical model: anagen onset is initiated by the activation of HFSCs in the hair germ, leading to the formation of transit-amplifying cells (TACs). Simultaneously, McSCs in the same niche are stimulated to proliferate and differentiate into mature melanocytes. The activities of HFSCs and McSCs are regulated by intrinsic and extrinsic factors (11). Intrinsic factors include the epigenetic landscape, genetic and transcriptional regulation within the bulge niche, and signals from the dermal papilla(DP), the central signaling hub for hair regeneration. Extrinsic factors involve influences from the microenvironment and macroenvironment. Modulating these microenvironments for HFSCs and McSCs may, therefore, promote hair regeneration and skin pigmentation (12). Disorders of HFSCs or McSCs may contribute to skin conditions such as hair loss, canities, and melanoma, and may also result in failure of repigmentation in conditions like vitiligo.

In vitiligo, melanocytes located in the interfollicular epidermis are the primary targets of destruction. However, melanocytes within hair follicles typically survive due to the IP present in this area. Differentiating hair follicle McSCs into functional melanocytes and directing them upward to the epidermis of vitiligo lesions presents a feasible approach for restoring normal pigmentation. If, however, the follicular melanocyte populations are destroyed, repigmentation of vitiligo lesions would not be possible (13). The primary pathogenic mechanism in both vitiligo and AA involves CD8 + T cell-mediated immune attacks. In vitiligo, these attacks primarily target interfollicular epidermal melanocytes located in the basal layer of the epidermis, though this immune assault can occasionally extend downward into the hair follicle, affecting melanocytes in the outer root sheath.

Anagen hair follicles of AA express major histocompatibility complex (MHC) class 1and class 2 and infiltrates of T lymphocytes are found in the deep dermis close to the hair bulb and perifollicular region (14). These phenomena indicate the collapse of immune privilege (15, 16). Then CD8 + T cells attack the anagen hair bulbs within the dermal layer (17) and damage the hair.

**FIGURE 2:**
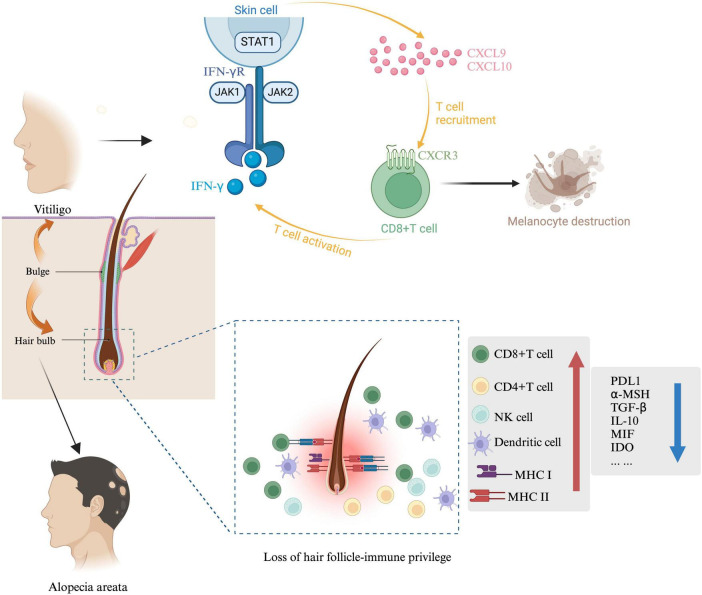
Immunological mechanisms in AA and vitiligo. McSC and HFSC are both present in the hair bulge, the sources of stem cells for both AA and vitiligo are indicated by arrows. In vitiligo, IFN-γ and IFN-γ-induced genes are predominant in lesional skin. IFN-γ is essential for recruiting melanocyte-specific, autoreactive CD8 + T cells to the skin. The IFN-γ-induced chemokine CXCL10 and its receptor CXCR3, expressed on autoreactive CD8 + T cells in the blood and lesional skin of vitiligo patients, are critical for T cell recruitment in vitiligo. In AA, collapse involves suppression of immunomodulatory factors such as TGF-β, PD-L1, IL-10, α-MSH, and MIF, along with increased MHC expression in hair follicles. MHC-I and MHC-II, crucial for immune cell antigen recognition, are reduced in the bulge and hair bulb during hair follicle IP. Other mediators produced by hair follicles, including neuropeptides (α-MSH), growth factors (TGF-β1/2, IGF-1), and MIF, also help suppress CD8 + T cells and NK cells, maintaining a stable hair growth cycle.

The hair follicle is an immune-privileged site, capable of preventing autoimmune reactions mediated by self-antigens. While the exact pathogenesis of AA remains unclear, a prevailing theory suggests it is closely associated with the breakdown of hair follicle IP (18). Firstly, factors such as increased secretion of IFN-γ, upregulation of NKG2D ligands (such as MICA and ULBP3/6), elevated expression of MHC I and MHC II molecules, chemokines (such as IL-15, IL-2, and CXCLs), and reduced levels of local “IP guardians” (such as TGF-β1, IL-10, α-MSH, IDO, VIP) collectively increase the exposure of follicular autoantigens. This exposure contributes to the breakdown of follicle IP and can trigger AA. Secondly, genetic factors also influence hair follicle vulnerability. Variants in genes expressed in the follicle, such as PRDX5 and STX17, may impair follicular IP, making it more susceptible to autoimmune reactions (19). According to Stephanie et al., KRT82 has been identified as a risk gene for AA, with 11 damaging variants, including missense and loss-of-function mutations, which potentially disrupt intermediate filament assembly and compromise the structural integrity of keratins in the hair shaft cortex (20). Thirdly, oxidative stress may further induce the upregulation of NKG2D ligands, contributing to the breakdown of IP and heightened autoimmunity (21). Fourthly, inflammation may drive the hair follicle into a malnourished growth phase and premature senescence. γδ T cells, predominantly distributed within and around the hair follicle, promote inflammation, which leads to elevated expression of NKG2D and IFN-γ, thus triggering AA (22). The primary goals of AA treatment are to inhibit disease progression and promote hair regrowth. The extent of hair loss and the patient’s age are critical factors in clinical management (Figure 2).

## 3 Hair follicle regeneration

### 3.1 Mechanism of hair follicle regeneration

The hair follicle is a complex structure within the skin, playing a critical role in both hair growth and wound healing (23). Hair regeneration is effectively initiated by activating HFSC proliferation and their migration downward into the hair matrix, promoting the transition of the follicle from the resting phase to the growth phase (24). McSCs, responsible for hair pigmentation, reside in the bulge and hair germ regions of telogen-phase hair follicles, where they coexist with epithelial stem cells and progenitor cells (25). HFSCs are regulated by various intrinsic and extrinsic signals, including Bone Morphogenetic Protein(BMP), Forkhead Box C1(FOXC1), Nuclear Factor of Activated T-cells, Cytoplasmic 1 (NFATC1), Sonic Hedgehog(Shh), and Wnt/β-Catenin(Wnt)pathways. Wnt and BMP signaling are particularly important in governing HFSC behavior and hair differentiation. In the HFSC lineage, quiescent HFSCs are activated by Wnt signals and BMP inhibitory factors. Once activated, epithelial Wnt signaling suppresses Sox9 expression, promoting progenitor cell differentiation (26). FOXC1 is essential for establishing multi-bulge hair follicle architecture; its loss may accelerate hair cycling and compromise long-term hair coat maintenance (27). NFATC1, primarily expressed in HFSCs within their niche, is activated by upstream BMP signaling and functions downstream to transcriptionally repress CDK4, thereby maintaining HFSC quiescence (28). Shh interacts antagonistically with the Wnt pathway, playing a key role in hair follicle development and regeneration (29). These pathways are essential for regulating HFSC behaviors. Additionally, the DP, comprising mesenchymal cells, governs hair follicle growth. Epithelial-mesenchymal interactions between DP and HFSCs are crucial for maintaining follicle morphogenesis and the hair growth cycle (23).

Vitiligo and AA are autoimmune skin disorders characterized by abnormal immune responses against skin cells and frequently co-occur. Investigating their shared genetic foundations is essential for uncovering underlying disease mechanisms and identifying potential therapeutic targets. In the GWAS risk loci for vitiligo and AA, rs2476601-A has been identified as a shared risk locus for both diseases. Summary-based Mendelian randomization has identified four common genes associated with these conditions: HLA-DRB6, HLA-DQA2, HLA-DRB1, and HLA-DQA1 (30). These shared genetic factors provide a scientific basis for understanding the similarities between the two disorders and may reveal therapeutic targets applicable to both. Further identification of genes or molecular pathways impacting follicular function could facilitate the development of combined treatment strategies for vitiligo and AA.

### 3.2 Sources of hair follicles

Traditional therapies for vitiligo and AA present certain limitations, including limited efficacy, potential side effects, high relapse rates, restricted treatment options, psychological impacts, and the absence of targeted therapies. Moreover, insufficient donor hair follicles challenge surgical interventions for individuals with extensive hair loss. In light of these limitations, researchers have explored the development of novel therapeutic approaches. Among the most promising is hair follicle regeneration, which is critical for treating both vitiligo and AA. However, the limited availability of follicle sources remains a constraint. Identifying several potential follicle sources may offer new insights into effective treatments (Table 1).

**TABLE 1:** The role of hair follicle regeneration in vitiligo and AA.

		Vitiligo	AA
Similarities	Pathogenesis	There exist common signaling pathways that regulate the activity of HFSCs and McSCs to achieve hair regeneration and pigmentation such as Wnt/β-catenin signaling, BMPs, PDGF, Follistatin, HGF, Angptl7, Angptl4, Shh signaling, Notch signaling.	
	The role of hair follicle	In both diseases, the original source of regeneration is the hair follicle bulge, which repopulates the vitiligo-depigmented epidermis with melanocytes, and supplies melanocytes and keratinocytes for the formation of new hairs in AA.	
	Treatment	Non-invasive: Corticosteroids, topical calcineurin inhibitors, narrowband UVB phototherapy, contact immunotherapy and stem cells. Invasive: Follicular Unit Extraction, Follicular unit transplant, Platelet-rich plasma(PRP)	
Difference	Pathogenesis	The affected area primarily lies in the bulge region of the hair follicle	The affected area primarily lies in the hair follicle bulb.
	The role of hair follicle	Stem cell bank for vitiligo repigmentation (containing melanocyte precursors capable of proliferating, migrating, and differentiating)	AA regenerates as intrafollicular regeneration: during the late telogen and early anagen phases of the hair follicle, the proliferative precursor of the bulge just above the dermal papilla is activated to form a new bulb
	Treatment	Mini-punch grafting(mPG) Trichiasis electrolyzer combined with single hair follicle transplantation	Scalp microneedle therapy Fractional radiofrequency

HFSCs and dermal papilla cells (DPCs) are crucial for effective hair restoration and regeneration (31). They are utilized in several therapeutic approaches for hair regeneration, including: (1) reversing pathological processes underlying hair loss (particularly in androgenetic alopecia), (2) regenerating entire hair strands from bulge-derived cells, and (3) creating new hair follicles through stem cell cultures or tissue engineering techniques (32). DPCs located in the cephalic region originate from the neural crest, while those in other areas derive from the mesoderm. Both freshly isolated and cultured DPCs inertact with neighboring epithelial HFSCs via complex signaling pathways, facilitating the induction of non-hair-bearing epidermis to form hair follicles (33). Therefore, DPCs, when expanded *in vitro*, represent an optional source of dermal cells for hair follicle regeneration (34).

Secondly, induced pluripotent stem cells (iPSCs), with their self-renewal capacity and potential to differentiate into cells from all three embryonic germ layers, are considered invaluable for bioengineering hair follicles (35). In recent years, the reprogramming of somatic cells into iPSCs through the forced expression of specific factors has emerged as an alternative approach for stem cell-based applications (34). Lee et al. demonstrated that hair follicles could be generated from iPSCs by enabling the self-organization of differentiating iPSCs into three-dimensional structures known as skin organoids (36). Approximately 70 days post-cultivation, hair began to emerge from these organoids, indicating successful complex skin morphogenesis. By 100 days, the skin organoids developed structures resembling those formed during embryonic skin development, including fat, neurons, and melanocytes surrounding the hair follicles. On day 140, skin organoids were implanted in the dorsal skin of nude mice, resulting in hair growth of 2–5 mm in 55% of the xenografts (37). This study is particularly promising as it presents a novel approach to hair follicle regeneration, facilitating both hair follicle regrowth and skin repair while modeling human *in vivo* development, thus providing a research platform with substantial potential.

Thirdly, mesenchymal stem cells (MSCs) are proliferative, multipotent cells found in most body tissues, with the capacity for self-renewal and differentiation into multiple cell types. MSCs possess two essential properties—immune evasion and immunomodulation—that make them attractive candidates for treating various inflammatory and immune-mediated diseases (38). Adipose-derived stem cells (ADSCs) were the first MSCs shown to stimulate hair growth through the secretion of several growth factors, including hepatocyte growth factor (HGF), vascular endothelial growth factor (VEGF), insulin-like growth factor (IGF), and platelet-derived growth factor (PDGF). As a result, adipose-derived stem cell (ASC) conditioned medium and ASC exosomes have been developed as cosmetic ingredients and are commercially available (39). In summary, ADSCs hold significant promise for promoting hair follicle growth and treating alopecia. Continued advancements in stem cell biology, regenerative medicine, and clinical applications are expected to drive the development of innovative and effective hair restoration therapies.

### 3.3 Clinical application of hair follicles in vitiligo and AA

#### 3.3.1 Clinical application of hair follicles in vitiligo

The pathogenesis of vitiligo is characterized by the absence of melanocytes in the basal layer of the epidermis or by the loss of melanin-producing function within melanocytes (40). Primary treatments for vitiligo include topical corticosteroids, topical calcineurin inhibitors, and narrowband UVB phototherapy (41). Although various treatments are available, therapeutic outcomes often remain unsatisfactory, particularly for patients with stable vitiligo (42).

Both hair follicles and the epidermis serve as potential sources for pigment cell production in skin tissue, offering theoretical advantages for vitiligo treatment. Autologous cultured melanocytes have already been used with some success in the treatment of vitiligo, as reported by Czajkowski et al. (43). Compared to epidermal melanocytes, hair follicle-derived melanocytes present distinct benefits for vitiligo management (44). Firstly, hair follicles contain a higher number of melanocytes. Secondly, these melanocytes are larger, exhibit more dendritic, and possess enhanced synthetic capabilities. Additionally, donor sites for hair follicle-derived melanocytes show a lower incidence of disease (45). Furthermore, preparing a suspension from hair follicles involves fewer steps, thereby addressing some limitations associated with epidermal cell transplantation (46). Follicular unit transplantation was first applied in 1998 for recoloring vitiligo plaques. Na et al. reported the successful transplantation of complete individual follicles into vitiligo lesions, with 71% of patients exhibiting pigment deposition around the transplanted follicles (47). Given clinical evidence suggesting the potential role of hair follicles in skin pigmentation, increasing numbers of researchers have proposed the use of follicles as a promising therapeutic approach for vitiligo (48).

Mini-punch grafting (mPG) has been successfully employed for the treatment of stable vitiligo for several years and is recognized as a cost-effective and straightforward method. In this process, melanocytes are separated from the basal membrane and adjacent keratinocytes, allowing them to proliferate, migrate, reorient their dendrites, and reconnect with surrounding cells to facilitate pigment diffusion (49). The primary reservoir of melanocytes resides within the hair follicles, particularly in the outer root sheath. Building on this, researchers have proposed a novel cell transplantation technique for vitiligo treatment using autologous non-cultured outer root sheath hair follicle cell suspension (50). In a study by Shi et al., skin grafts containing intact hair follicles were utilized as a melanocyte source. The cell suspension included not only melanocytes but also hair matrix components, dermal papillae, keratinocytes, and various other skin cell types. Among 26 stable vitiligo patients unresponsive to conventional treatments, 22 achieved over 75% re-pigmentation, with 9 patients achieving more than 90% re-pigmentation (50). Thus, autologous hair follicle cell transplantation represents a promising new option for vitiligo patients who do not respond to standard therapies.

#### 3.3.2 Clinical application of hair follicles in AA

First-line treatments for patchy AA include topical and intralesional corticosteroids. Corticosteroid therapies for AA can be administered topically, intralesionally, or systemically to reduce local inflammation and suppress immune attacks on hair follicles (51). However, the effectiveness of topical corticosteroids may be limited, as follicular inflammation around hair follicle bulbs is often located 4–7 mm beneath the skin’s surface (52). Intralesional corticosteroids, injected directly into AA lesions to achieve high local drug concentrations, produce a stronger immunosuppressive effect at the hair loss site (53).

The level of inflammation in AA is significant, rendering direct hair growth-promoting drugs largely ineffective when used alone. Therefore, a more effective treatment approach may involve targeting specific components of the immune system or its interactions with dysfunctional anagen-stage hair follicles (54). Minoxidil is employed in AA treatment, typically as an adjunct to an immunosuppressive agent. A recent review of multiple studies reported that 57% of patients receiving minoxidil as adjunctive therapy experienced hair regrowth. Notably, the success rate increased to 85% when patients were treated with a combination of oral minoxidil and an oral Janus kinases (JAKs) inhibitor (55).

JAKs are essential tyrosine kinases in cytokine signaling, regulating immune cell functions such as proliferation and differentiation. By blocking the JAK/STAT pathway, JAK inhibitors can reduce cytokine production associated with AA, including IFNγ, thereby decreasing inflammation targeting hair follicles (56). Although pro-inflammatory cytokines are critical for immune signaling, they may also inhibit hair growth (18). Baricitinib, an oral selective JAK inhibitor, has been used in the treatment of severe AA in adults. In a recent study, Brett King found that down-titrating baricitinib from 4 mg to 2 mg maintained a clinical response in over half of the patients with severe AA, although a higher rate of sustained response was observed in patients who remained on the 4 mg dose (57). In another study led by Maryam Nasimi, among 97 AA patients treated with oral tofacitinib for at least 6 months, 69.1% demonstrated an improvement of over 50% in their SALT (severity of alopecia tool) scores, with 44.3% achieving a SALT score reduction of 90% or more, suggesting that tofacitinib is an effective and safe treatment option (58). Studies have also shown that both tofacitinib and ruxolitinib significantly promote hair growth; however, hair growth induced by ruxolitinib tends to be of shorter duration compared to tofacitinib (59). Additionally, current research indicates that ruxolitinib, baricitinib, and tofacitinib are effective treatments for vitiligo (60). Given the shared pathogenic mechanisms between AA and vitiligo, JAK inhibitors hold potential for treating comorbid cases, potentially providing dual therapeutic benefits. Further research could investigate whether these inhibitors effectively target overlapping immune pathways, offering insights into new treatment strategies for autoimmune diseases.

Follicular unit transplantation, which involves two methods of follicle acquisition: follicular unit transplantation (FUT) and follicular unit extraction (FUE) (61) is a surgery popularly known for its utility in androgenetic alopecia (AGA) (62). Hair transplantation in AA is not practiced as the implanted follicular grafts might be destroyed due to underlying autoimmune pathology. However, Kerure et al., successfully did FUE in AA patients. Hair growth was achieved within 6 months with no recurrence during 1-year follow up (63).

Compared to the conventional treatments currently used in clinical practice and emerging immunotherapies, surgical treatment for AA remains in the clinical trial stage. However, it holds promise as a potential therapeutic approach. Autologous hair follicles may be more suitable for hair transplantation due to the absence of immune rejection. However, in cases of limited availability, the use of three-dimensional (3D) skin models has also attracted attention. Hair follicular reconstitution relies on highly organized epithelial-mesenchymal interactions. In 2019, Seung Hwan Paik and colleagues developed a 3D skin equivalent model using neonatal mouse epidermal and dermal cells, and it was shown that this model could reliably regenerate hair follicles (64).

The pathophysiology of AA is complex, as localized disease manifestations often mask the underlying systemic immune activity. In treating AA, therapies that directly target hair follicles, alongside immunosuppressive agents, may be particularly valuable. Strategies focused on extending the anagen phase of hair follicles, reducing immune-mediated follicular damage, and promoting follicular repair and regeneration represent promising directions for future AA treatments.

## 4 Prospects and challenges

Significant advancements have been made in the research on vitiligo and AA. The skin’s immune system typically maintains homeostasis, granting immune privilege (IP) to skin cells. Disruption of this immune balance can lead to a loss of IP, rendering skin cells and their appendages vulnerable to immune cell attacks. The IP of hair follicles is thought to play a protective role in AA, though whether this IP extends to depigmented hair in vitiligo lesions warrants further investigation.

In summary, the pathogenic mechanisms of vitiligo and AA are complex. Historically viewed as distinct skin disorders, mounting evidence suggests a correlation between these two autoimmune diseases. Hair follicles have emerged as a critical focal point in the study of both conditions, providing valuable insights into their pathogenesis and potential therapeutic interventions. Hair follicle melanocyte stem cells (McSCs) display a dual role, supporting hair regeneration when migrating downward and promoting melanocyte generation when migrating upward. Wound-induced hair neogenesis offers a new perspective for treating both diseases, as appropriate injury can stimulate the Wnt/β-catenin signaling pathway, inducing both hair regeneration and pigment deposition. However, novel immune therapies targeting hair follicles and the technique of trauma-induced hair follicle neogenesis have yet to undergo large-scale clinical trials, leaving their efficacy and safety uncertain. Hair follicle transplantation for treating AA and vitiligo also has limitations. Firstly, treatment outcomes remain unpredictable, influenced by factors such as individual variability, disease severity, and autoimmune response. Secondly, follicle regeneration requires long-term therapy; achieving optimal results often demands extended observation and treatment, with ongoing medication or surgical intervention, which adds complexity, cost, and invasiveness. Thirdly, adverse reactions, including local inflammation, infection, autoimmune rejection, or uneven pigmentation, may arise, affecting patients’ daily lives and reducing treatment acceptability. Fourth, donor follicles are generally limited to specific regions, such as the occipital scalp, restricting this approach for patients with extensive vitiligo or AA, particularly for those with stable or non-active AA. Further research is encouraged to explore these areas, particularly focusing on the shared aspects of hair follicles in both conditions. This approach holds promise for developing more effective and patient-centered treatment strategies.
